# Comparison of Sun Protection Factor (SPF) 30 Persistence Between Inorganic and Organic Sunscreen in Swimmers: Protocol for a Multicenter, Randomized, Noninferiority, Split-Body, Double-Blind Clinical Trial

**DOI:** 10.2196/42504

**Published:** 2022-12-21

**Authors:** Karin Rachmani, Shannaz Nadia Yusharyahya, Adhimukti Sampurna, Respati W Ranakusuma, Sandra Widaty

**Affiliations:** 1 Department of Dermatology and Venereology, Faculty of Medicine Cipto Mangunkusumo General Hospital Universitas Indonesia DKI Jakarta Indonesia; 2 Clinical Epidemiology and Evidence-Based Medicine Unit, Faculty of Medicine Cipto Mangunkusumo Hospital Universitas Indonesia DKI Jakarta Indonesia

**Keywords:** inorganic sunscreen, organic sunscreen, persistence, sun protection factor, sunscreen, swimmer, swimming

## Abstract

**Background:**

Outdoor swimming athletes are often exposed to undesirable environmental conditions such as long-term sun exposure. The risk of sunburn can still occur in this population due to the loss of sunscreen and an increase in the sensitivity of the skin to ultraviolet rays, particularly ultraviolet B, in wet conditions. Some previous trials showed that organic sunscreens had a longer shelf-life than inorganic sunscreens after exercise due to their characteristics to bind better with the skin layer. Meanwhile, inorganic sunscreens tend to form layers on the skin’s surface so that they can be more easily removed. To our knowledge, no studies evaluate sunscreens' resistance, either inorganic or organic, after exercising in Indonesia.

**Objective:**

This study aims to evaluate the persistence of inorganic versus organic sunscreens used by swimmers. The primary objective is to assess whether the inorganic sunscreen is as good as the organic sunscreen in the field of the persistence of sunscreens after swimming for 1.5 hours.

**Methods:**

This study is a randomized, split-body, double-blind, noninferiority, and multicenter clinical trial in Cikini, Jakarta, Indonesia. An estimated 22 athletes in each group, who aged 18-40 years and practice in the morning or afternoon, will be randomized using a computer-generated randomization method. We calculated the sample size using the difference in the average decrease in sun protection factor (SPF) levels that is considered significant based on the clinical judgment set by the researchers, which was 5. Neither the research subjects nor the researchers are aware of the type of sunscreen that will be applied. The hypothesis will be tested using paired-sample t test or Wilcoxon to assess the difference of SPF levels in each group between organic and inorganic sunscreens with SPSS (version 20.0; IBM Corp).

**Results:**

This study has been approved by the Ethical Committee Faculty of Medicine Universitas Indonesia and is funded by the International Publication Grant from Universitas Indonesia. The enrollment process was completed in December 2020.

**Conclusions:**

This study will test all procedures in preparation for conducting the main study, including several potential obstacles and challenges from the perspective of participating physicians and eligible swimmers. The study results will be disseminated through publications in a peer-reviewed journal with Open Access format. This study will provide information about SPF 30 persistence in sunscreens and the best type of sunscreen to be used while swimming, particularly for athletes.

**Trial Registration:**

ClinicalTrials.gov NCT04618536; https://clinicaltrials.gov/ct2/show/NCT04618536?term=NCT04618536

**International Registered Report Identifier (IRRID):**

RR1-10.2196/42504

## Introduction

Athletes who train and compete for outdoors such as in swimming pools are often exposed to undesirable environmental conditions, such as excessive humidity, hot and cold weather, windy conditions, and long-term sun exposure. These exposures cause some skin conditions [1]. In Indonesia, the average training time of an athlete is 5 times a week for 1.5-2 hours per day. Exercise is carried out in the morning and evening, when the ultraviolet index (UVI) was in the range of 1-4, meaning that taking shelter, wearing closed clothes such as hats, using sunscreen, and other sun protection manners should be done [2].

When swimmers train in an outdoor swimming pool, apart from being exposed to the pool water, they are also exposed to sun’s radiation. Water could wash away the applied sunscreen, increasing the risk of photosensitivity [3]. In addition, there will be an increase in humidity in the stratum corneum, which functions to protect the skin from ultraviolet (UV) radiation [4,5].

In order to prevent sunburn, sun protection is needed, which can be achieved in several ways, such as using a sunscreen [4]. There are 2 types of sunscreens used based on the filter component, specifically organic and inorganic sunscreens. Organic sunscreen absorbs and prevents UV light to enter the epidermis; meanwhile, inorganic sunscreen works by reflecting and scattering radiation [6-8].

Some previous trials showed that organic sunscreens had a longer shelf-life than inorganic after exercise due to its characteristics to bind better with the skin layer. Meanwhile, inorganic sunscreens tend to form a layer on the skin’s surface, so that it can be more easily removed. Until this time, there have been no previous studies regarding the resistance of sunscreens, either organic or inorganic, after exercising in Indonesia. Therefore, this study aimed to evaluate the persistence of sunscreen with a sun protection factor (SPF) 30 used by swimmers after 1.5 hours.

## Methods

### Study Aims and Objectives

First, this study aims to assess whether inorganic sunscreen is as good as organic sunscreen in the field of the persistence of sunscreens after swimming for 1.5 hours. SPF of inorganic and organic sunscreens will be calculated before and after swimming training. The difference between those times will be measured and compared. SPF will be quantified using the minimal erythema dose (MED) test that will be conducted in 2 days. Irradiation will be carried out on the first day, and MED results will be collected 24 hours after irradiation.

Second, we assess the SPF value resulting from in vivo method conducted before swimming. The SPF of either organic or inorganic sunscreens will be compared in the manner of MED. This trial also aims to know the decreasing level of SPF after swimming for 1.5 hours and which type of sunscreen provides higher persistence.

### Study Design and Setting

This is a randomized, split-body, double-blind, noninferiority, multicenter clinical trial. The recruiting center is Cikini swimming pool, located in Jakarta, Indonesia. Recruitment began in August 2020 and ended in December 2020.

Research subjects were selected from Cikini swimming center in Jakarta, Indonesia. Data collection will be carried out on the same person using the split-body method. Each research subject will receive 2 treatments in the form of inorganic and organic sunscreens. The sunscreen application will be executed on the back area on the right and left for each treatment simultaneously.

### Eligibility Criteria

Inclusion criteria are as follows: female or male swimming athletes aged 18-40 years, practicing swimming at least 3 times a week with a duration of 1.5-2 hours per practice in the morning or afternoon, willing to be the subject of research by signing the consent form, do not have skin diseases or a history of allergies to sunscreens.

Exclusion criteria were as follows: existence of skin lesions in the test area; undergoing phototherapy; using drugs with photosensitivity side effects; history of skin malignancy, photosensitivity reactions, or disease affected by UV rays; exposure to direct sunlight to the test area 24 hours before the study and during the study period; absence of an erythema response 24 hours after the radiation test; and erythema occurs in the entire test area box 24 hours after the radiation test.

### Interventions

This study used a sunscreen made by PT Paragon Technology and Innovation. Both sunscreens are made in the form of an oil-in-water emulsion with the addition of a film-forming layer to maintain their resistance to water. Both organic and inorganic sunscreens have been tested using a simulator to determine their SPF levels.

Interventions will be conducted according to research manual of operations (Multimedia Appendix 1). First, we will mark the area on the back. In each area, we will draw 6 circles as locations for the irradiation test. We will apply as much as 2 mg/cm^2^ sunscreen on each area within 2 stages, particularly at the first and second sessions. The sunscreen will be applied using a 1-cc syringe to cover all areas. After that, the sunscreen is spread using gloves, starting with circular and then followed by horizontal and vertical movements with light pressure. During the smearing process, the smearing finger remains in contact with the skin for 35 seconds ±5 seconds. Gloves are changed at each smearing of a different test area.

**Figure 1:**
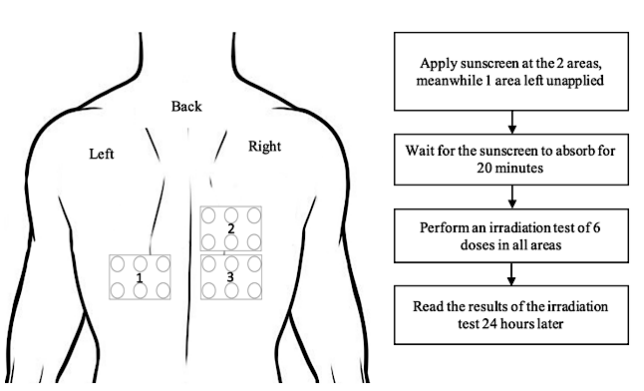
Study procedure at the first meeting.

At the first meeting, the back is marked with 3 areas (Figure 1). Sunscreens are applied to 2 areas; meanwhile, 1 area is left without any sunscreen applied. After 20 minutes, the irradiation test is performed. The MED values were calculated 24 hours after irradiation to determine the SPF of each sunscreen. This procedure is carried out to take note of the basic data and the suitability of the SPF with data that are listed on the packaging.

**Figure 2:**
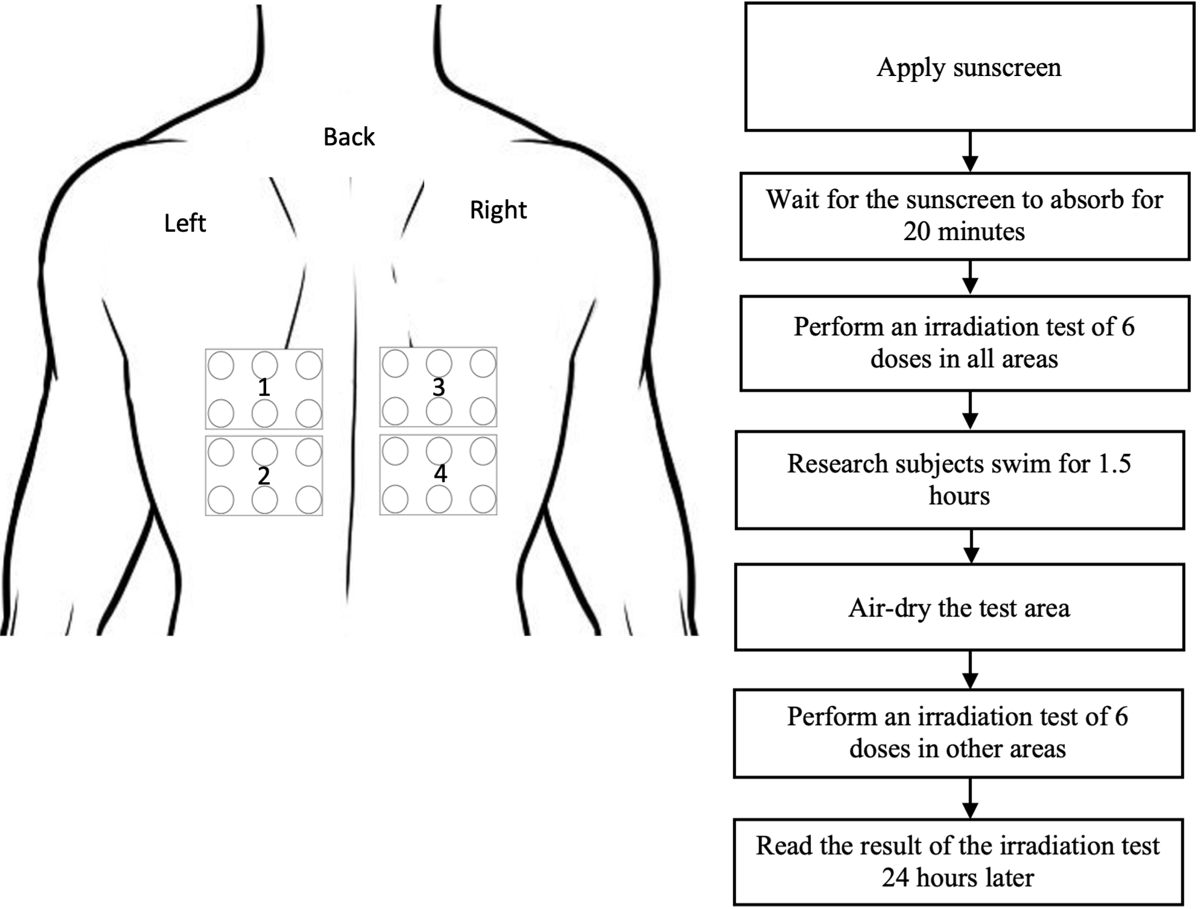
Study procedure at the second meeting.

The second meeting will be conducted 1 week after the first one. At the second session, the back is marked with 4 areas (Figure 2). Both types of sunscreens are applied to all areas, where 2 areas are for the test before swimming and the other 2 areas are for the test after swimming. After 20 minutes, an irradiation test is performed. Athletes were then asked to carry out the exercise for 2 hours. After completion, the athletes were asked to dry their body without using a towel. The MED values were calculated 24 hours after irradiation to determine and compare the SPF of each sunscreen before and after the swimming period. Swimming activities are carried out in the morning or evening when the UVI is in the range of 0-2.

The SPF measurement of pre- and postswimming was obtained using an in vivo method adapted with modifications from ISO 24444 in 2019 and COLIPA 2006 by bringing the instrument to the study site and in a room with a temperature of 18-26 °C. The back area will be marked into 3 sections measuring 8 cm×5 cm each. Each area will be marked with a 6×3 cm^2^ perforated sticker according to The Daavlin Lumera probe.

### Discontinuation of Study Medication

The study medication must be discontinued if a suspected anaphylactic reaction or there are serious adverse events on the MED test square such as pain and formation of blisters during its administration or if the patient participation consent is withdrawn.

### Standard Treatment for the Management of Adverse Events

If side effects occur in the form of blisters, pain, edema, or bright red erythema after irradiation, the research subjects will be treated with normal saline compresses for 15 minutes twice a day. The compresses can be continued with topical corticosteroids twice a day after bathing. Research subjects who experienced side effects will be excluded from the study, but their development will continue to be followed until they recover.

### Outcomes

This study will provide mean differences in SPF by 4 to establish noninferiority with a 95% lower confidence limit. The hypothesis will be tested using paired samples *t* test or Wilcoxon test to assess the difference of SPF levels in each group between organic and inorganic sunscreens. The difference in SPF level will be declared to be no different if the *P* value for the paired *t* test or Wilcoxon test is >.05, and the upper limit of the CI does not exceed 4 SPF level. The persistence of organic and inorganic sunscreens is assessed using the following parameters: (1) the MED is defined as the dose of UV radiation that induces just perceptible erythema on exposed skin 24 hours after irradiation. This MED result will be stated in mJ/cm^2^. (2) SPF is defined as the ratio of MED between protected and unprotected skin areas. The average SPF value from each type of sunscreen will be computed. The SPF value will be rounded up to one decimal in the index unit. (3) Persistence of SPF is defined as the lowest differences of SPF value before and after 1.5 hours swimming, stated in index unit.

### Participant Timeline

**Figure 3:**
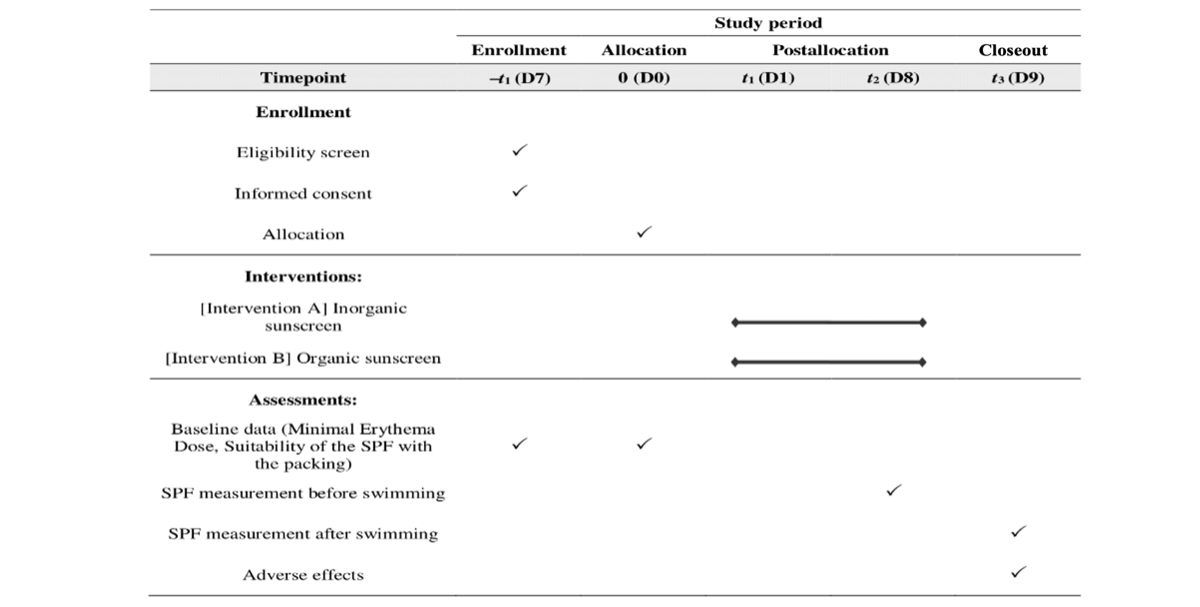
The schedule of enrollment, interventions, and assessments. SPF: sun protection factor.

Participants will be directed according to the timeline in Figure 3. The enrollment of study participants will take 7 days from the eligibility screen until the allocation. There will be 2 measurement sessions in this study with a time interval of 7 days. The SPF measurement for each session will be carried out 24 hours after the session.

### Sample Size Estimates

The calculation of the sample size was carried out using the continuous variable noninferiority test formula and the comparison of the mean of the 2 groups in pairs. From the 2 formulas, the largest number of samples was taken for further use in research. Based on the previous clinical trial and risk of dropout, we estimated a minimum sample size of 22 participants in each group. We calculated the sample size using the difference in the average decrease in SPF levels that is considered significant, based on the clinical judgement set by the researchers (ie, 5). In total, 22 experimental subjects and 22 control subjects will be adequate to reject the null hypothesis that the population means of the experimental and control groups are equal with a probability (power) of 0.9. The type I error probability associated with this test of this null hypothesis is 0.05.

### Recruitment

Researchers will conduct COVID-19–related health protocols when collecting data in the context of preventing the pandemic spread. The body temperature and oxygen saturation of the research subjects will be checked before the examination. Each subject will be given a mask and face shield and asked to wash their hands. Researchers will provide the instructions to maintain a distance of at least 1 m during data collection. We will limit the number of subjects, only 4 athletes per day, to attend each sampling session. Before and after the test of each research subject, the tools will be cleaned using an alcohol swab.

### Allocation of Sequence Generation and Concealment Mechanism

Treatment allocation is applied by computer-based randomization [9] to determine the back area and type of sunscreen provided [10]. The allocation data for each subject will be placed in a nontransparent, sealed envelope along with sunscreen. At the time of data collection, the envelope will be opened, and sunscreen will be applied by the research assistant. Both randomization and treatment allocation will be carried out by statisticians who were not known by the researchers or the research subjects. The data will not be accessed until data collection for all subjects is completed.

### Blinding

Treatment will be allocated by numbering the research subjects and including the right-back area as number 1 and the left-back area as number 2. We will use computer-generated randomization method [9] to determine the back area and the type of sunscreen to be given [10]. Neither the research subjects nor the researchers will be aware of the type of sunscreen that will be applied. The allocation data for each subject will be placed in a sealed opaque envelope. At the time of data collection, the envelope will be opened, and the sunscreen will be applied by the research assistant. Both randomization and treatment allocation will be carried out by statisticians. The data will not be accessed until data collection for all subjects is completed. The irradiation test and the assessment of results will be carried out by researchers.

### Data Collection Methods

Before data collection begins, a preliminary study will be conducted to determine the value of broadband ultraviolet B (BB-UVB) MED on various skin types as well as to conduct inter-reviewer reliability tests. The data will be obtained from the literature and the tool guide and used as a source of information on the differences in the test doses upon irradiation. Therefore, a preliminary study will be conducted to equalize the dose of the irradiation test. Reliability testing will be performed to achieve same perception to assess erythema to ensure the quality of the data produced during the research. The test will be carried out by showing 11 skin photos to 3 assessors to determine the MED of each photo [11]. After that, the readings of the 3 assessors will be compared based on the intraclass correlation (ICC). An ICC value close to one, in this study set at 0.9, indicates that the reviewer has the same understanding of the MED reading so that, in the study, the MED reading can be carried out by the researcher.

We will use inclusion and exclusion criteria to select the research subjects. We will provide explanations about the purpose and research method before research subjects sign a consent form. There will be 2 sessions in the study: the first one for basic data collection and research sample selection and the second one for providing treatment and executing the randomization process.

After the consent form is signed, we will conduct history taking, physical examination, and documentation. History of systemic disease, skin disease, daily activities, malignancies, and family history will be recorded. We will assess the skin type and identify any skin lesion on physical examination.

This study will use metal halide UV enhanced lamp BB-UVB in the active spectrum of 290-320 nm (The Daavlin Lumera) for UVB radiation test. The device will be calibrated before every data collection.

### Data Management and Statistical Methods

All data obtained on the research status will be recorded for further coding. The collected data will be analyzed using SPSS (version 20.0; IBM Corp). The analysis will be carried out in 2 stages: descriptive and inferential analyses. In descriptive analysis, each variable is explained according to the type of data. The distribution of numerical data is assessed by looking at the normality value. The distribution of normal data is displayed with the mean and SD values. Categorical data are displayed in the form of frequency and percentage tables. In the inferential stage, the hypothesis will be tested using paired *t* test or Wilcoxon test to assess the differences in the decrease in SPF levels in each group of inorganic and organic sunscreens, and between the 2 groups. We used a 1-sided CI approach in the statistical analysis. The mean difference in SPF will be no different if the *P* value for the paired *t* test is >.05, and the upper limit of the CI does not exceed 4 SPF.

### Data Monitoring

Since this is a short study, it does not require a data monitoring committee. The process and quality of patient recruitment, data entry, and a compilation of research data in the main database will be supervised by an independent assessor from the Clinical Epidemiology and Evidence-Based Medicine unit of Dr. Cipto Mangunkusumo Hospital (CMH), Faculty of Medicine Universitas Indonesia (FMUI), who is not involved in this study. Physicians will identify, evaluate, and handle any cases of major adverse events. These cases will be recorded and reported to, as well as be reviewed by, the Medical Ethics Committee Faculty of Medicine University of Indonesia.

### Harms

Contrary to adverse effects or adverse drug reactions, which are all unpleasant and unintended responses to a study drug related to any dose, an adverse event is defined as any untoward medical occurrence in a patient or clinical investigation subject administered a pharmaceutical product and which does not necessarily have to have a causal relationship with the study medication. After the eligible participants sign the written agreement and enroll themselves in the study, adverse events and adverse drug reactions will be gathered. All negative events that take place after study enrollment, throughout additional treatment, or during hospitalization owing to negative occurrences or negative reactions will be documented.

Any untoward medical occurrence at any dose that may result in inpatient or prolonged hospitalization, persistent or significant disability, medically significant events, life-threatening events, or death is considered a serious adverse event, and the subject will receive adequate treatment in addition to being recorded and reported to the Medical Ethics Committee of the FMUI. Unless there is a temporal relationship between the study medications or another protocol procedure and the events, or if the event is unexpected or unexplained given the subjects' clinical course, previous medical conditions, and concomitant medications, we will not report serious adverse events that occur after the study is discontinued. The serious adverse event form will contain a record of every serious adverse incident.

### Auditing

We will create an audit committee from the Clinical Epidemiology and Evidence-Based Medicine unit of the Dr. Cipto Mangunkusumo Hospital, Faculty of Medicine Universitas Indonesia, which is separate from the research investigators of the main study. The International Conference Harmonization-Good Clinical Practice standards and the protocol will always be followed when observing and evaluating the study's quality.

### Access to Data

The cleansed data sets will be accessible to the primary investigator. The primary investigator will also be able to request and have direct access to each site's data sets. Passwords will be used to safeguard the project data sets. Data will be distributed to project team members blinded of any participant identifying information in order to ensure confidentiality.

### Ethics Approval

This clinical trial has been registered into ClinicalTrials.gov with identifier NCT04618536 and approved by the Clinical Research Ethics Committee of Faculty of Medicine Universitas Indonesia ID numbers 20-09-1037. Study results will be disseminated through peer-reviewed publications in the Open Access format.

## Results

This study is funded by International Publication Grant from Universitas Indonesia. The enrollment process was completed in December 2020 and data analysis was conducted in January-March 2021. The study result is expected to be completed in January 2022 and submitted for publication in February 2022.

## Discussion

We developed a protocol for a randomized, split-body, double-blind, noninferiority clinical trial to evaluate the persistence of the inorganic versus organic sunscreens in the outdoor swimmers with estimated 22 participants in each group. This study aims to assess whether inorganic sunscreen is as good as organic sunscreen in the field of the resistance of sunscreens after swimming for 1.5 hours of training. The secondary objectives are SPF value resulting from in vivo method, which types of sunscreens provide higher persistence.

COVID-19 health protocol during the pandemic will be conducted. Before data collection begins, we will conduct a preliminary and inter-reviewer reliability testing. The instrument will be calibrated first before use at each session of data collection. All subjects had to sign the consent form if they are willing to participate. Irradiation test will be done and then followed by SPF measurement of the sunscreens developed by PT Paragon Technology and Innovation in the form of an oil-in-water emulsion. The study medication must be discontinued if suspected adverse events occur or if patient consent for participation is withdrawn. Research subjects will be excluded from the study, but their recovery will be followed by researchers.

The collected data will be analyzed using SPSS (version 20.0; IBM Corp). This clinical trial has been approved by the ethic committee and registered to ClinicalTrials.gov. Study results will be disseminated through publications in the Open Access format. This study will provide information about SPF 30 persistence in sunscreens and the best type of sunscreen to be used while swimming, particularly to athletes.

## Appendix

Multimedia Appendix 1 Manual of operation as a summary of the study procedures for the research team.

Multimedia Appendix 2 Peer reviewed by University of Indonesia Q3 PUTI Grant / Hibah PUTI Q3 Universitas Indonesia Tahun 2020 (Jakarta, Indonesia).

## Abbreviations

BB-UVB: broadband ultraviolet B
ICC: intraclass correlation
MED: minimal erythemal dose
SPF: sun protection factor
UV: ultraviolet
UVI: ultraviolet index

## Data Availability

The data sets analyzed during the current study are available from the corresponding author on reasonable request.
