# Supplementary material for: Comparison of Sun Protection Factor (SPF) 30 Persistence Between Inorganic and Organic Sunscreen in Swimmers: Protocol for a Multicenter, Randomized, Noninferiority, Split-Body, Double-Blind Clinical Trial
Source: JMIR Res Protoc. 2022 Dec 21;11(12):e42504. doi: 10.2196/42504 (PMC9813813; doi:10.2196/42504)
Supplement: Multimedia Appendix 1 [file resprot_v11i12e42504_app1.docx]

**Manual of Operations**

**Comparison of Sun Protection Factor (SPF) 30 Persistence between Organic and Inorganic Sunscreen in Swimmers: A Multicenter, Randomized, Non-Inferiority, Split-Body, Double Blind Clinical Trial Protocol**

Rachmani, Karin; Yusharyahya, Shannaz; Sampurna, Adhimukti; Ranakusuma, Respati Wulansari; Widaty, Sandra

**Research summary**

Athletes who train and compete outdoor such as swimming pools are often exposed to undesirable environmental conditions, such as excessive humidity, hot and cold weather, windy conditions, and long-term sun exposure. These exposures cause some skin conditions.[1] In Indonesia, the average training time of an athlete is 5 times a week for 1,5-2 hours per day. Exercise is carried out in the morning and evening, when the ultraviolet index (UVI) was in the range of 1-4 meaning that taking shelter, wearing closed clothes such as hats, using sunscreen, and other sun protection manners should be done.[2] When swimmers train in an outdoor swimming pool, apart from being exposed to the pool water, they are also exposed to sun radiation. Water could wash away the applied sunscreen increasing the risk of photosensitivity.[3] In addition, there will be an increase in humidity in the stratum corneum which functions to protect the skin from UV radiation.[4-6]

In order to prevent sunburn, sun protection is needed which can be achieved in several ways, such as using sunscreen.[5,7] There are two types of sunscreen based on the filter component, specifically organic and inorganic. Organic sunscreen absorbs and prevents UV light to enter the epidermis, meanwhile, inorganic sunscreen works by reflecting and scattering radiation.[8] Some previous trials showed that organic sunscreens had a longer shelf-life than inorganic after exercise due to its characteristics to bind better with the skin layer. Meanwhile inorganic sunscreens tend to form layer on ther skin’s surface, so that it can be more easily removed. Until this time, there have been no previous studies regarding the resistance of sunscreens, either organic or inorganic, after exercising in Indonesia, so we conducted this randomized, split-body, double-blind, noninferiority, and multicenter clinical trial located at Jakarta, Indonesia.

The objectives of this study are to: (1) assess whether inorganic sunscreen is as good as organic sunscreen in the field of the persistance of sunscreens after swimming for 1.5 hours and (2) assess the SPF value resulting from in vivo method, conducted before swimming. If inorganic sunscreens prove to be effective and high of the persistance to UV radiation, they could become useful to the swimming athletes to prevent sun exposure-related skin disease in this population.

**References**

1. Blattner CM. Dermatological conditions of aquatic athletes. World J Dermatologyy [Internet]. 2015;4(1):8. Available from: http://www.wjgnet.com/2218-6190/full/v4/i1/8.htm

2. Petry S. Intersun: The global UV project: A guide and compendium [Internet]. World Health Organization. 2003. 54 p. Available from: https://search.ebscohost.com/login.aspx?direct=true&db=bth&AN=116652501&site=ehost-live&scope=site

3. De Luca JF, Adams BB, Yosipovitch G. Skin manifestations of athletes competing in the summer olympics: What a sports medicine physician should know. Sport Med. 2012;42(5):399–413.

4. Moehrle M, Koehle W, Dietz K, Lischka G. Reduction of minimal erythema dose by sweating. Photodermatol Photoimmunol Photomed. 2000;16(6):260–2.

5. Lautenschlager S, Wulf HC, Pittelkow MR. Photoprotection. Lancet. 2007;370(9586):528–37.

6. Harrison SC, Bergfeld WF. Ultraviolet light and skin cancer in athletes. Sports Health. 2009;1(4):335–40.

7. Wang SQ, Balagula Y, Osterwalder U. Photoprotection: A review of the current and future technologies. Dermatol Ther. 2010;23(1):31–47.

8. Shaukat S, Aman S, Kazmi AH. Skin and sports. J Pakistan Assoc Dermatologists. 2015;25(3):206–10.

**Step-by-step procedures**

**Health protocol in COVID-19 pandemic**

**Objective(s)**

To ensure safe conditions in data collection in the context of preventing the COVID-19 spread.

**Tool(s)**

1. Thermometer
2. Oxymeter
3. Medical mask
4. Face shield
5. Hand sanitizer
6. Alcohol swab

**Personnel**

Participating physicians as research assitants

**Procedures**

1. The body temperature and oxygen saturation of the research subjects will be checked before we started the examination.
2. Each subject will be given a mask and face shield and asked to wash their hands using hand sanitizer provided from researchers.
3. Researchers will provide the instructions to maintain a distance of at least 1 meter during data collection.
4. We will limit the number of subjects, only 4 athletes per day, to attend each sampling session. The irradiation test will be carried out in a closed room. When the test is done, the number of people in the room is limited to a maximum of 3 people. Everyone in the test room is required to wear a mask and face shield.
5. Before and after the test of each research subject, the tools will be cleaned using an alcohol swab. Research subjects who are waiting their turn will wait outside the examination room and sit on chairs 1 meter apart from one another.

**Preliminary research and inter-reviewer reliability testing**

**Objective(s)**

To determine the value of broadband ultraviolet B (BB-UVB) minimal erythemal dose (MED) on various skin types as well as to conduct inter-reviewer reliability tests. The data which is obtained from the literature and the tool guide will be used as a source of irradiation showed differences in the test dose. Therefore, a preliminary study was be done to equalize the dose of the irradiation test.

**Tool(s)**

1. Single lens reflex camera (Canon^®^ EOS 7D) for skin photos

**Personnel**

1. Participating physicians as research assitants
2. Dermatologists as assessors of the MED

**Procedures**

1. Reliability test will be executed to achieve same perception to assess erythema to ensure the quality of the data produced during the research.
2. The test will be carried out by showing 11 skin photos to 3 assessors to determine the MED of each photo.
3. After that, the readings of the MED of each photo from the three assessors will be compared based on the intraclass correlation (ICC).
4. ICC value close to one, in this study set at 0.9, indicates that the reviewer has the same understanding of the MED reading so that in the study, the MED reading can be carried out by the researcher. This value is statistically significant.

**Tools calibration**

**Objective(s)**

To minimise any measurement uncertainty by ensuring the accuracy of the test equipment.

**Tool(s)**

1. The Daavlin Lumera^®^

**Personnel**

Participating physicians as research assitants

**Procedures**

This study used The Daavlin Lumera® with metal halide UV enhanced lamp BB-UVB in the active spectrum of 290-320 nm. The device will be calibrated before data collection.

**Study consent, recruitment, and randomisation**

**Objective(s)**

1. Identify the eligibility of potential athletes to be included in the trial
2. Provide adequate information concerning the research, including the process and potential adverse effects caused by the study
3. Obtain the consent from eligible patients to participate or not to participate in the study
4. Allocate the participant to become either intervention or control group randomly

**Tool(s)**

1. Participating information sheet and consent form
2. Study registration form
3. Eligibility form
4. Non-transparant and sealed envelopes
5. Computer or smartphone with internet connection for accessing the randomisation website

**Personnel**

Participating physicians as research assitants

**Procedures**

1. The participating physician will confirm the eligibility of the patient using inclusion and exclusion criteria. If the athlete has responded “Yes” to all inclusion criteria and “No” to all exclusion criteria, then the athlete is eligible for the study.
2. The physician will explain the purpose, research method, and risk of adverse effects of this study orally based on the participating information sheet.
3. The physician then obtain the consent for the trial using consent form. The athlete will be informed regarding sessions of this study. There were two sessions in the study, namely the first session for basic data collection as well as research sample selection, and the second session for providing treatment and executing the randomisation process.
4. Randomisation will begin by numbering the research subjects and including the right back area as area number 1 and the left back as area number 2.
5. Treatment allocation is applied by computer-based randomisation (www.randomizer.org) to determine the back area and type sunscreen provided.
6. The allocation data for each subject was placed in a non-transparent and closed envelope along with sunscreen.
7. At the time of data collection, the envelope will be opened and the application of sunscreen is done by the research assistant.
8. Both randomization and treatment allocation were carried out by statisticians and were not known by the researchers or the research subjects. The data will not be opened until data collection for all subjects is completed.

**Collecting baseline data and examination**

**Objective(s)**

To obtain relevant information and perform the basic physical dermatology examination

**Tool(s)**

1. Head lamp
2. Gloves

**Personnel**

Participating physicians as research assitants

**Procedures**

1. After the consent form is signed, we will do the history taking, physical examination and documentation.
2. History of systemic disease, skin disease, daily activities, malignancies, and family history will be recorded.
3. We will assess the skin type and identified any skin lesion on physical examination.

**Irradiation test and SPF measurement**

**Objective(s)**

1. To perform the irradiation test
2. To measure the SPF of the organic and inorganic sunscreens applied in this study

**Tool(s)**

1. The Daavlin Lumera^®^
2. UV protection glasses
3. Test sticker
4. Organic Sunscreen SPF 30
5. Inorganic sunscreen SPF 30
6. Gloves
7. Syringe 1 cc
8. SLR cameras and tripods
9. Non-fluorescent ring light
10. Blue background cloth

This study will use inorganic and organic sunscreens made by PT Paragon Technology and Innovation that has been formulated according to the research needs. Both sunscreens are made in the form of oil in water emulsion with the addition of a film forming layer. The inorganic and organic sunscreens have been tested using a simulator to determine their respective SPF levels.

**Personnel**

Participating physicians as research assitants

**Procedures**

1. We will mark the area on the back. In each area, we drew six circles as locations for the irradiation test. We applied as much as 2 mg/cm^2^ sunscreen on each area within two stages, particularly at the first and second sessions.
2. At the first session:
   1. The back is marked with three areas.
   2. Organic and inorganic sunscreens will be applied in two areas; while one area is left without sunscreen.
   3. After 20 minutes of applying sunscreen, irradiation is carried out. MED values will be calculated 24 hours after irradiation in each group to determine the SPF of each sunscreen. This is done to find out the basic data and the suitability of the SPF with what is listed on the package.


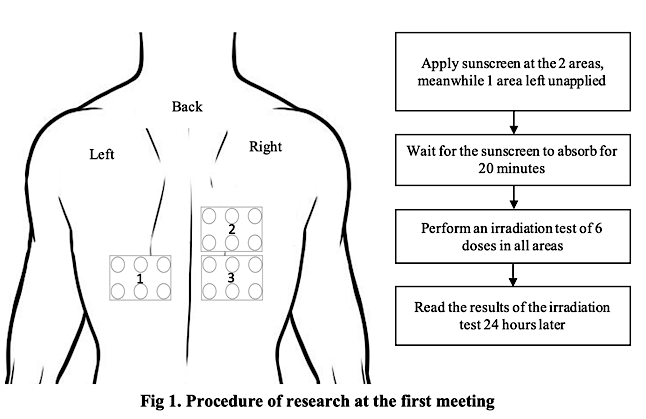


1. At the second session:
   1. This session will be conducted one week after the first irradiation test.
   2. The back is marked with four areas.
   3. Organic and inorganic sunscreens are applied to four areas; two areas to do the irradiation test before and two areas to do the irradiation test after swimming.
   4. After 20 minutes of applying sunscreen, irradiation is carried out.
   5. Athletes are then will be asked to do the exercise for 1,5 hours. Swimming activities are carried out in the morning or evening when the UVI is in the range of 0-2.
   6.
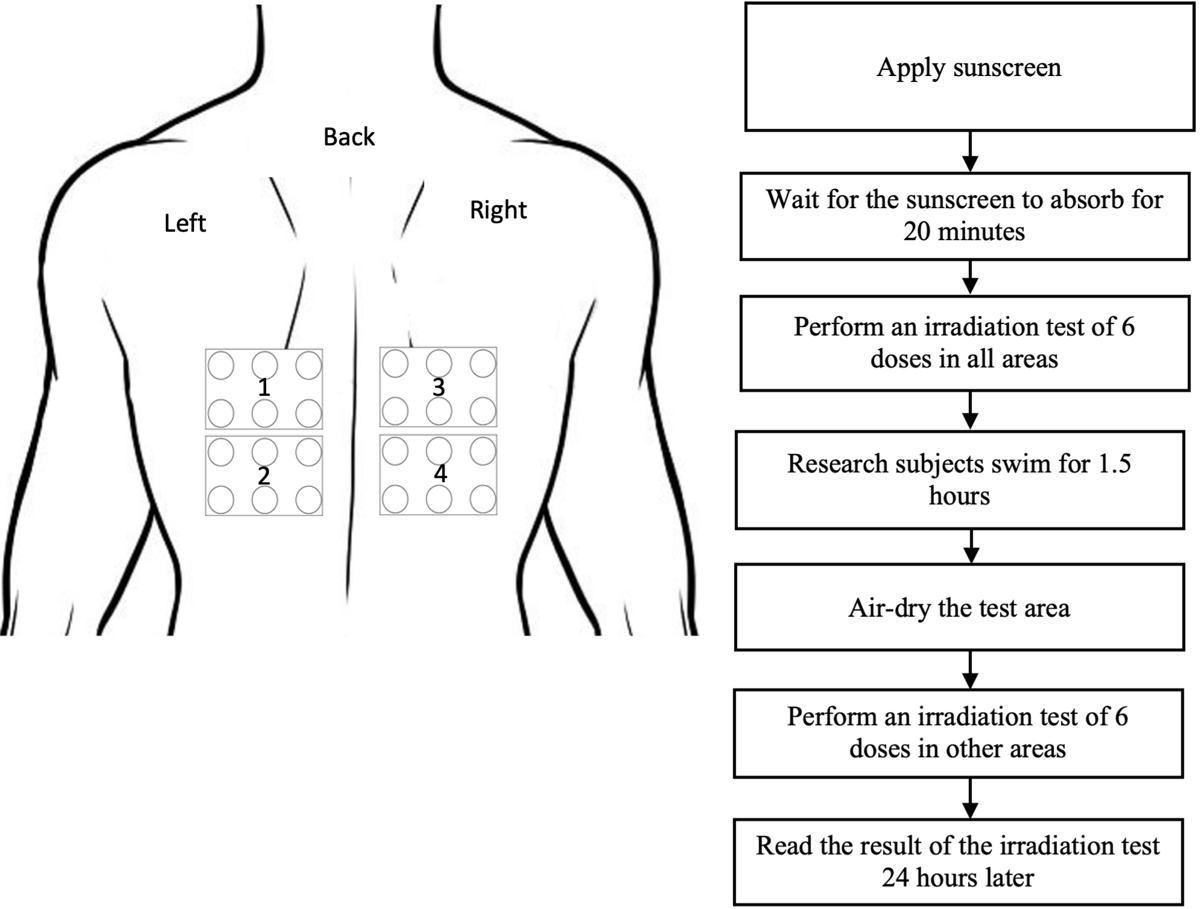
After completion, the athletes will be asked to dry the body without using a towel (air dry), then irradiated on two other areas that has been applied inorganic and organic sunscreen. MED values were calculated 24 hours after irradiation in each group to determine the SPF of each sunscreen before and after swimming.

**Fig 2. Procedure of research at the second meeting**

1. The SPF examination will be carried out using an in vivo method adapted with modifications from ISO 24444 in 2019 and COLIPA 2006. The examination will be done by bringing the instrument to the research location and in a room with a temperature of 18-26^o^C. The SPF calculation are carried out as follows:
   1. Selecting research subjects according to the criteria.
   2. Research subjects change clothes with clothes that have been provided in the changing rooms that have been prepared.
   3. Research subjects are seated sequentially according to the research number.
   4. Determine the skin type of the research subject based on the criteria of Fitzpatrick skin type.
   5. Give the research label number on the subject's back after confirming the identity and irradiation number.
   6. Mark the area to be tested, namely the back area between the waist and the scapula. The back area will be marked into three sections measuring 8 cm x 5 cm each. The three areas consist of one area that is not applied sunscreen (unprotected) and two areas that are applied sunscreen (protected). Each area will be marked with a six-3 cm^2^ perforated sticker.
   7. Apply sunscreen on the tested area at a dose of 2 mg/cm2 (+0.05 mg/cm2) using gloves. The application begins with placing sunscreen on the skin using a 1 cc syringe to cover all areas uniformly. After that, the sunscreen is spread on the skin area using gloves; starting with circular movements and followed by horizontal and vertical movements with light pressure. During the smearing process, the smearing finger remains in contact with the skin for 35 seconds + 5 seconds. Gloves will be changed at each smearing of a different test area.
   8. Wait for the sunscreen to absorb for 20 minutes.
   9. Instruct the use of UV-protective goggles on research subjects and operators who are indoors.
   10. Provide UVB exposure to the marked area.
   11. The irradiation process will be carried out with the position of the research subject sitting with his back to the operator. In the unprotected area, the UVB dose is increased gradually by 1.25 in each circle. In the protected area, the irradiation time is varied by multiplying the unprotected MED by the expected SPF. The dose is increased gradually by 1.25 based on the expected value of SPF 30. The duration of exposure was based on the timetable on the instrument according to the last calibration.
   12. Mark the six irradiation areas with a skin marking pen and remove the sticker.
   13. Remove sunscreen on the test area using a cotton swab that has been moistened with micellar water.
   14. Educate research subjects not to sunbathe, not to apply anything in the test area, and not to take new drugs.
   15. Assess MED in the test area 24 hours after irradiation. The assessment is carried out in a covert manner. Researchers who assessed the erythema response in each study subject were different from those who applied sunscreen.

**Assessing and managing adverse events**

The physician will record and inform the adverse events to the all investigator, particularly to those with serious or severe adverse events. The study medication must be discontinued if a suspected anaphylactic reaction or there are serious adverse events on the MED test square such as pain and formation of blister during its administration or if the patient participation consent is withdrawn.

If side effects occur in the form of blisters, pain, edema or bright red erythema after irradiation, the research subjects will be treated with 0.9% NaCl compresses for 15 minutes, twice a day. Compresses can be continued with topical corticosteroids twice a day after bathing. Research subjects who experience side effects are excluded from the study, but their development will continue to be followed until they recover.

**Appendix 1. Patient allocation flow chart**

Athletes aged 18-40 years

Participant selection:

Inclusion and exclusion criteria

Baseline data and examination:

- Evaluate MED
- Assessing the suitability of the SPF with the packaging

Exclusion criteria

Inclusion criteria

- There are skin lesions in the test area.

- In phototherapy treatment.

- Using drugs with photosensitivity SE.

- History of skin malignancy, history of photosensitivity reactions or history of diseases affected by UV rays.

- Exposure to direct sunlight on the test area or sunbathing 24 hours before the study and during the study period.

- There was no erythema response 24 hours after the irradiation test.

- There is erythema all over the test area box 24 hours after the test.

- Female or male swimmers aged 18-40 years.

- Practice swimming at least 3 times a week with a duration of 1.5-2 hours per time in the morning or afternoon.

- Willing to be a research subject.

- Have no skin disease.

- No history of allergy to sunscreen.

Research subjects

1 week

Randomisation

**Appendix 2. Data collection flow chart**

Data collection with intervention grup:

Organic sunscreen

SPF measurement before swimming

Swimming activities for 1,5 hours

Result

SPF measurement after swimming

- Side effects
- Refuse to continue research

Drop out

Inorganic sunscreen

Data analysis and report
