# Supplementary material for: Comparison of Sun Protection Factor (SPF) 30 Persistence Between Inorganic and Organic Sunscreen in Swimmers: Protocol for a Multicenter, Randomized, Noninferiority, Split-Body, Double-Blind Clinical Trial
Source: JMIR Res Protoc. 2022 Dec 21;11(12):e42504. doi: 10.2196/42504 (PMC9813813; doi:10.2196/42504)
Supplement: Multimedia Appendix 2 [file resprot_v11i12e42504_app2.pdf]

UNIVERSITAS INDONESIA
Veritas, Probitas, Iustitia

Kampus Salemba Jl. Salemba Raya No. 4, Jakarta 10430
Kampus Depok Gedung Pusat Administrasi Universitas
Kampus Universitas Indonesia Depok 16424
T. 62.21. 7867 222/ 7884 1818 **F.** 62.21. 7884 9060
E. pusadmui@ui.ac.id | **www.ui.ac.id**

NOTA DINAS
Nomor : ND-923/ UN2.RST/PPM.00.00/2022

Yth. : Dr. dr. Sandra Widaty, SpKK(K)
Dari : Direktur Riset dan Pengembangan
Perihal : Laporan Hasil Reviu Proposal Riset Hibah PUTI Q3 Universitas Indoensia Tahun 2020

Bersama surat ini kami sampaikan hasil reviu proposal riset Hibah PUTI Q3 Universitas Indonesia Tahun 2020 atas nama Dr. dr. Sandra Widaty, SpKK(K) dengan judul *"Perbandingan Ketahanan Sun Protection Factor 30 Tabir Surya Organik dan Inorganik Setelah Aktivitas Renang"*, dinyatakan telah **lolos seleksi substansi** berdasarkan reviu yang dilakukan oleh tim reviewer.

Demikian surat ini kami sampaikan. Atas bantuan dan kerja sama Ibu, kami ucapkan terima kasih.

3 Oktober 2022
Direktur Riset dan Pengembangan

Munawar Khalil, S.Si., M.Eng.Sc., Ph.D.
NUP100111610232806891

UNIVERSITAS INDONESIA
Veritas, Probitas, Iustitia

Kampus Salemba Jl. Salemba Raya No. 4, Jakarta 10430
Kampus Depok Gedung Pusat Administrasi Universitas
Kampus Universitas Indonesia Depok 16424
T. 62.21. 7867 222/ 7884 1818 **F.** 62.21. 7884 9060
E. pusadmui@ui.ac.id | **www.ui.ac.id**

Lampiran Nota Dinas Nomor : ND-923/ UN2.RST/PPM.00.00/2022

Hasil Reviu Proposal Riset Hibah PUTI Q3 Universitas Indonesia Tahun 2020

Komentar :	1. Metode design tidak jelas, sehingga perlu penjelasan lebih lanjut terkait design yang digunakan<br>2. Apakah menggunakan design eksperimen?<br>3. Adakan kontrol dalam penelitian?<br>4. Pada proposal hanya ada prosedur intervensi, apakah ada prosedur lainnya?<br>5. Perlu menambahkan kriteria inklusi dan eksperimen pada proposal
